# Acute Binge Alcohol Consumption Presenting With a Cytotoxic Lesion of the Corpus Callosum (CLOCC) in a Young, Healthy Filipino Male: A Case Report With Features Overlapping the Proposed Marchiafava-Bignami Disease Spectrum

**DOI:** 10.7759/cureus.113680

**Published:** 2026-07-30

**Authors:** Neil Anthony C Sarto, Jon Stewart H Dy, Jasmyn A De Leon

**Affiliations:** 1 Neurology, Chinese General Hospital and Medical Center, Manila, PHL

**Keywords:** binge alcohol consumption, cytotoxic lesion of the corpus callosum, diffusion-weighted imaging, magnetic resonance imaging, marchiafava–bignami disease, splenium of the corpus callosum, stroke mimic

## Abstract

Marchiafava-Bignami disease (MBD) is a rare toxic-metabolic encephalopathy characterized by corpus callosum involvement, classically associated with chronic alcoholism and malnutrition. Cytotoxic lesions of the corpus callosum (CLOCCs) represent a radiologic pattern that may occur in various metabolic, toxic, and infectious conditions and can mimic acute ischemic stroke. We report a 28-year-old previously healthy Filipino male who presented with an acute, remittent, and moderately severe bi-occipital headache following an episode of acute binge alcohol consumption. Systemic and neurological examinations were unremarkable. Cranial magnetic resonance imaging (MRI) revealed diffusion restriction in the splenium of the corpus callosum, initially interpreted as acute ischemic infarction, while magnetic resonance venography was normal. The patient was managed with antiplatelet therapy, citicoline, and analgesics. Extensive diagnostic evaluation, including transesophageal echocardiography, cranial computed tomography angiography, carotid duplex ultrasonography, 24-hour Holter monitoring, and cerebrospinal fluid analysis, yielded unremarkable results. Repeat cranial MRI performed 10 days after discharge demonstrated marked regression of the splenial lesion. The patient remained asymptomatic, with no neurological deficits, and was fully independent in activities of daily living. This case highlights an acute, reversible CLOCC triggered by binge alcohol consumption, presenting as a stroke mimic within the MBD spectrum. Recognition of this entity is essential to avoid misdiagnosis and unnecessary long-term antithrombotic therapy. To our knowledge, this represents the first locally documented case of this presentation in the Philippines.

## Introduction

Marchiafava-Bignami disease (MBD) is a rare toxic-metabolic encephalopathy characterized by primary involvement of the corpus callosum and is classically associated with chronic alcohol consumption and malnutrition [1]. Cytotoxic lesions of the corpus callosum (CLOCCs), in contrast, represent a radiologic pattern of transient callosal injury that may occur in association with diverse metabolic, toxic, infectious, inflammatory, and other systemic conditions [2,3]. Although CLOCCs and MBD may share overlapping neuroimaging features, they are not synonymous entities, and the relationship between them remains incompletely understood. Neuroimaging in MBD may demonstrate lesions involving the corpus callosum, which can occasionally manifest as CLOCCs, particularly within the splenium [2]. These radiologic findings may closely mimic acute ischemic infarction, posing a diagnostic challenge [3].

Although MBD has traditionally been described in the setting of long-standing alcoholism, increasing evidence suggests that acute metabolic or toxic insults may produce reversible callosal lesions within the same disease spectrum [4]. However, cases associated with acute binge alcohol consumption in previously healthy individuals remain rarely reported [4], and there has been no local documentation of such presentations in the Philippines.

We report the case of a young Filipino male who presented with an acute headache following alcohol intoxication and was initially diagnosed with ischemic infarction of the splenium of the corpus callosum on magnetic resonance imaging. Subsequent investigations were unremarkable, and follow-up neuroimaging demonstrated complete resolution of the lesion within 10 days, consistent with a reversible cytotoxic callosal lesion triggered by acute alcohol exposure.

## Case presentation

A 28-year-old, previously healthy Filipino male was admitted to our institution with a 10-day history of an acute, remittent, and moderately severe bi-occipital headache following an episode of acute binge alcohol consumption involving beer and cocktails. The headache was aggravated by coughing, temporarily relieved by analgesics, and associated with intermittent low-grade fever and throat discomfort. There was no associated vomiting, visual disturbance, limb weakness, sensory symptoms, altered mental status, or seizures. He denied illicit drug use and had no known medical comorbidities.

On systemic examination, the patient was afebrile and hemodynamically stable. An oropharyngeal examination revealed bilaterally enlarged, hyperemic, and exudative tonsils. Neurological examination demonstrated an intact sensorium, with no evidence of cortical dysfunction, cranial nerve palsy, motor or sensory deficits, cerebellar signs, meningeal signs, or pathological reflexes.

Initial laboratory investigations, including complete blood count, serum electrolytes, renal and liver function tests, inflammatory markers, and coagulation profile, were within normal limits except for slightly elevated C-reactive protein (Table 1).

**Table 1:** Laboratory tests done during admission Legend: g/dL = grams per deciliter; mm3 = cubic millimeters; fL = femtoliter; mg/dL = milligrams per deciliter; mmol/L = millimoles per liter; U/L = units per liter

Test	Results	Reference Value
Hemoglobin	13.4	11.6-15.5 g/dL
Hematocrit	37.8	36.0-47.0%
White Blood Cell Count	5,200	4,800-10,800 cells/mm^3^
Neutrophils	54	40-74%
Lymphocytes	34	19-48%
Monocytes	10	3-9%
Platelet Count	225,000	150,000-400,000 cells/mm^3^
Mean Corpuscular Volume	83.3	82-98 fL
Mean Corpuscular Hemoglobin	29.5	28-33 pg
Mean Corpuscular Hemoglobin Concentration	35.4	32-38%
Creatinine	0.71	0.55-1.2 mg/dL
Blood Urea Nitrogen	11.4	9-23 mg/dL
Sodium	137	136-145 mmol/L
Potassium	3.7	3.5-5.1 mmol/L
Magnesium	1.7	1.6-2.6 mmol/L
Chloride	102	98-107 mmol/L
Bicarbonate	25.6	20.0-31.0 mmol/L
Alanine Aminotransferase	26	10-49 U/L
Aspartate Aminotransferase	31	0-34 U/L
Glycohemoglobin	5.5	<5.7%
C-reactive Protein	4.09	<1.0 mg/dL
Procalcitonin	0.06	<0.1 mg/dL
Anti-nuclear Antibodies	Negative	Negative
D-dimer	0.27	<0.5 ug/mL

**Figure 1:**
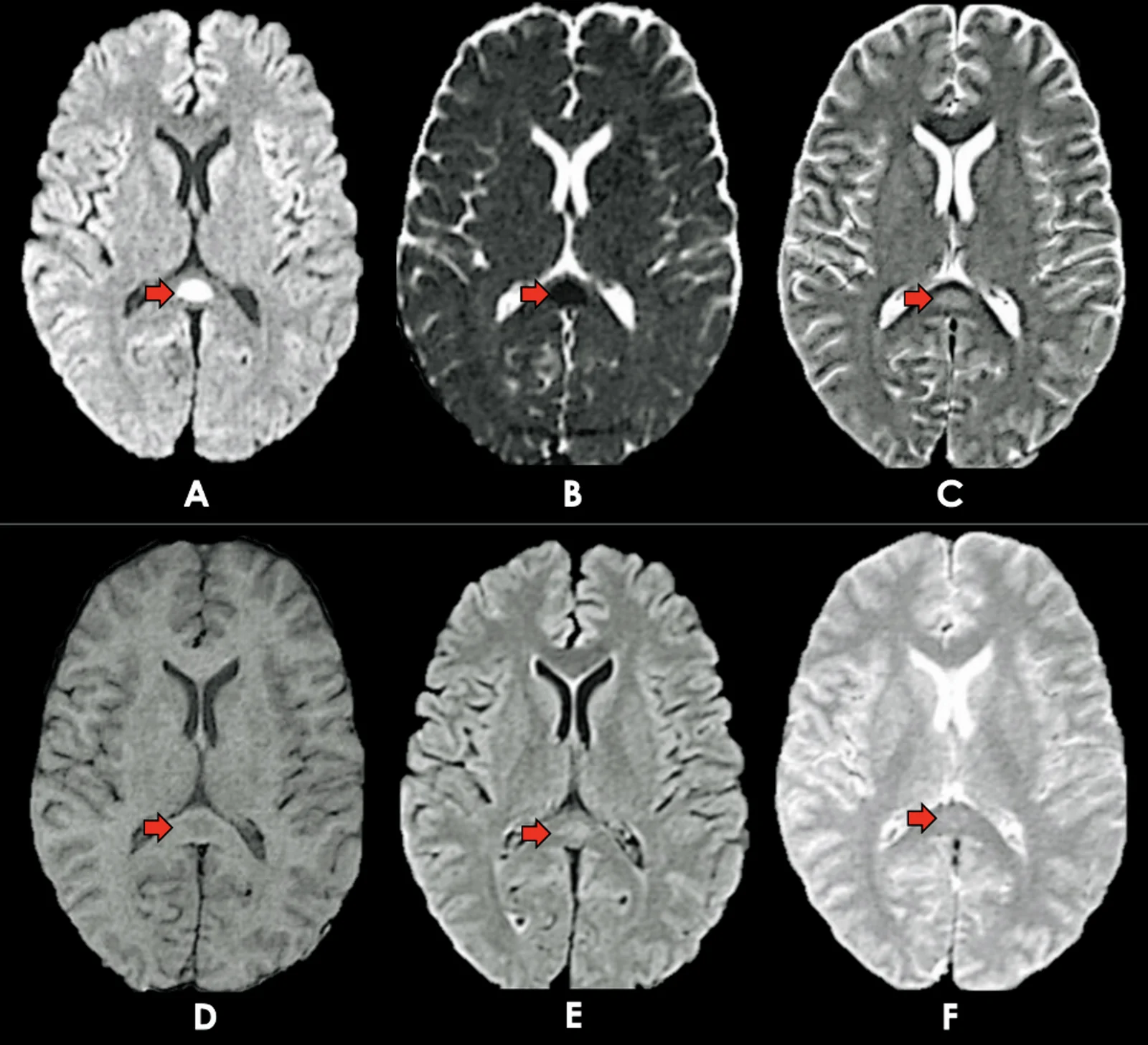
Initial cranial MRI demonstrating a focal cytotoxic lesion involving the splenium of the corpus callosum (arrow) (A) Diffusion-weighted imaging (DWI) demonstrates marked diffusion restriction. (B) Corresponding apparent diffusion coefficient (ADC) map shows low signal intensity, consistent with cytotoxic edema. (C) Axial T2-weighted image demonstrates subtle hyperintensity within the splenium. (D) Axial T1-weighted image demonstrates mild hypointensity within the splenium. (E) Axial fluid-attenuated inversion recovery (FLAIR) image demonstrates subtle hyperintensity within the splenium. (F) Gradient-recalled echo (GRE) sequence demonstrates no evidence of hemorrhage. MRI = magnetic resonance imaging

Given the patient’s new-onset persistent headache with red-flag features, including cough aggravation and associated low-grade fever, cranial magnetic resonance imaging (MRI) with magnetic resonance venography (MRV) was requested to evaluate for secondary causes of headache. MRI demonstrated diffusion restriction involving the splenium of the corpus callosum (Figures 1A, 1B), with corresponding T2-weighted and fluid-attenuated inversion recovery (FLAIR) hyperintensities (Figures 1C, 1E). Contrast-enhanced sequences showed no leptomeningeal or parenchymal enhancement. MRV revealed no evidence of cerebral venous thrombosis. Computed tomography angiography (CTA) of the head and neck demonstrated no large-vessel occlusion, arterial stenosis, or vascular malformation.

Further diagnostic evaluation to assess for cardioembolic, infectious, inflammatory, and autoimmune etiologies included transesophageal echocardiography and 24-hour Holter monitoring, all of which were unremarkable. Cerebrospinal fluid analysis showed normal opening pressure, normal cell count, and normal protein and glucose levels, with no evidence of infection or inflammation (Table 2).

**Table 2:** Cerebrospinal fluid analysis cm H2O = centimeter water; mm3 = cubic millimeters; mg/dL = milligrams per deciliter

Test	Results	Reference Value
Opening Pressure	8	8-18 cm H2O
Closing Pressure	3	8-18 cm H2O
Gross Description	Clear and free-flowing	Clear and free-flowing
Cells (Total)	1.11	0-5 cells/mm^3^
Red Blood Cells	0	0-5 cells/mm^3^
White Blood Cells	1.11	0-5 cells/mm^3^
Lymphocytes	100%	-
Neutrophils	0%	-
CSF glucose	3.24	2.2 - 3.9 mmol/L
Protein	43.3	15 - 45 mg/dL
Gram Stain	Negative	Negative
Bacterial Culture and Sensitivity	Negative at 3 days	Negative
Japanese B encephalitis virus, Dengue virus, SARS-CoV-2	Negative	Negative
Acid-fast Bacilli Smear	Negative	Negative
Mycobacterium tuberculosis Polymerase Chain Reaction	Negative	Negative
Mycobacterium tuberculosis Culture and Sensitivity	Negative for 42 days	Negative
Potassium Hydroxide	Negative	Negative
Cryptococcal Antigen Latex Agglutination System	Negative	Negative
India Ink	Negative	Negative
Escherichia coli K1; Haemophilus influenzae; Listeria monocytogenes	Negative	Negative
Neisseria meningitidis; Streptococcus pneumoniae	Negative	Negative
Streptococcus agalactiae; Cryptococcus neoformans	Negative	Negative
Cytomegalovirus; Enterovirus; Herpes Simplex Virus 1	Negative	Negative
Herpes Simplex Virus 2; Human Herpesvirus 6	Negative	Negative
Human Parechovirus; Varicella-Zoster Virus	Negative	Negative
Oligoclonal Bands	Negative	Negative
Cell Block	Negative	Negative
Cytology	Negative	Negative

The leading differential diagnosis was a CLOCC, given the isolated splenial involvement, absence of focal neurological deficits, and complete radiologic resolution on follow-up imaging. The temporal relationship with acute binge alcohol consumption favored a toxic-metabolic trigger, although a concurrent upper respiratory tract infection remained a plausible alternative precipitant.

Acute ischemic infarction of the corpus callosum was initially considered because of the diffusion restriction on MRI. However, the absence of focal neurological deficits, unremarkable CTA, MRV, and cardioembolic investigations, together with complete radiologic resolution within 10 days, made ischemia unlikely.

Infectious or inflammatory encephalitis was also considered because of the patient’s headache and low-grade fever. However, intact mental status, absence of seizures or focal deficits, normal cerebrospinal fluid findings, and lack of parenchymal or leptomeningeal enhancement argued against encephalitis. Demyelinating disease was considered less likely owing to the isolated splenial lesion, absence of multifocal lesions or clinical relapses, normal cerebrospinal fluid findings, and complete radiologic resolution.

Alcohol-related toxic-metabolic callosal injury, including possible Marchiafava-Bignami disease, remained within the differential diagnosis. However, the absence of chronic alcoholism, malnutrition, and diffuse corpus callosum involvement favored a possible alcohol-related CLOCC with overlapping features of the proposed MBD spectrum rather than definitive classic MBD.

The patient was initially managed with clopidogrel 75 mg once daily because acute ischemic infarction could not be excluded based on the initial MRI findings demonstrating isolated diffusion restriction within the splenium of the corpus callosum. He also received citicoline 1 g twice daily, analgesics for symptomatic relief of headache, and supportive care during hospitalization. Following comprehensive diagnostic evaluation and repeat MRI demonstrating regression of the lesion consistent with a reversible cytotoxic lesion of the corpus callosum, clopidogrel was discontinued because long-term antiplatelet therapy was no longer indicated.

**Figure 2:**
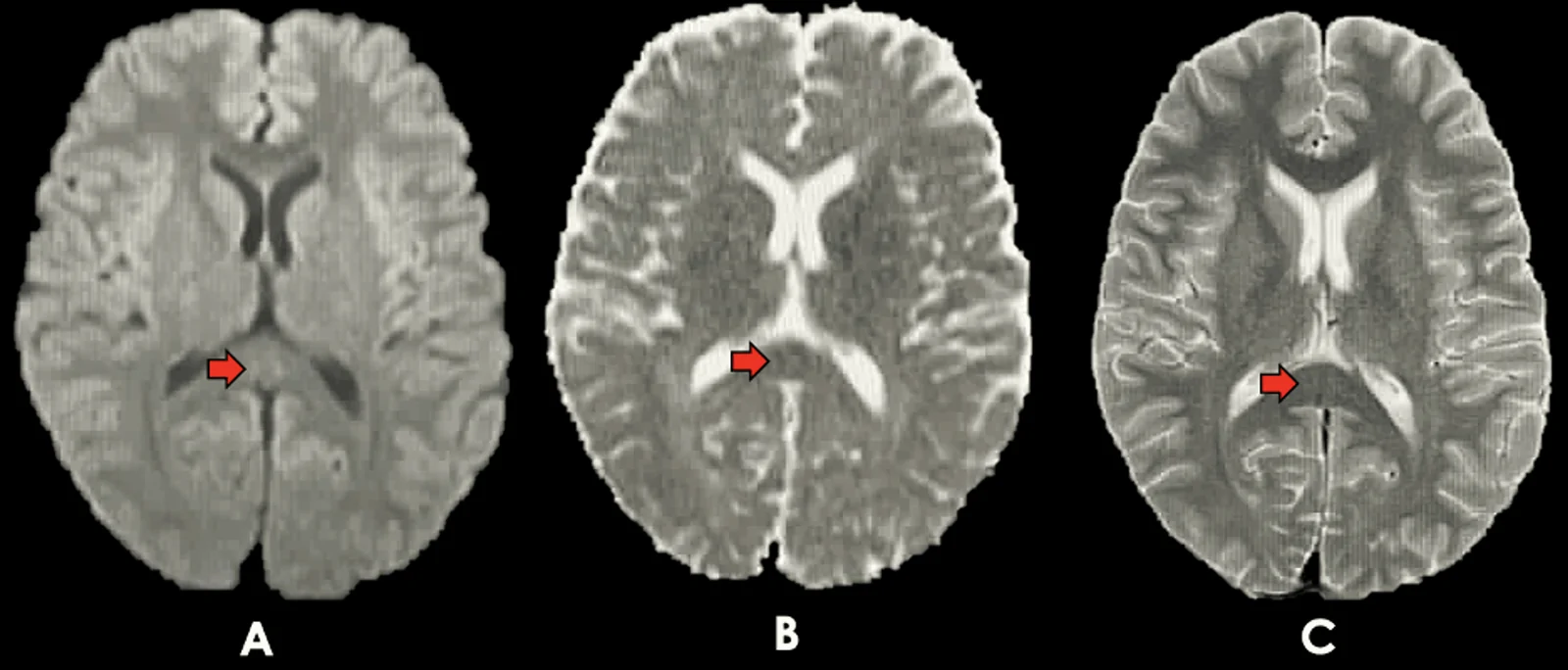
(A) Axial diffusion-weighted imaging (DWI) demonstrates complete resolution of the previously noted diffusion restriction in the splenium of the corpus callosum, with (B) normalization of signal on the apparent diffusion coefficient (ADC) map. (C) Axial T2-weighted sequences show resolution of the previously observed splenial hyperintensity. No new parenchymal signal abnormalities, hemorrhage, or abnormal enhancement are identified. MRI = magnetic resonance imaging

The patient’s hospital course was uncomplicated. His headache gradually improved throughout hospitalization, and he remained clinically stable with no focal neurological deficits. He was discharged with mild residual headache. Repeat cranial MRI performed 10 days after discharge (Figure 2) demonstrated significant regression of diffusion restriction with normalization of T2-weighted and FLAIR signal abnormalities within the splenium. At the three-month follow-up, repeat cranial MRI was unremarkable, with complete resolution of all previously identified imaging abnormalities. Clinically, the patient was asymptomatic, had no neurological deficits, and remained fully independent in all activities of daily living.

## Discussion

CLOCCs represent a distinctive radiologic pattern characterized by transient diffusion restriction, most commonly involving the splenium [3]. These lesions are increasingly recognized as important stroke mimics and have been reported in association with a wide range of metabolic, toxic, infectious, and inflammatory conditions [5-7]. The underlying pathophysiology is thought to involve cytotoxic edema mediated by excitotoxic injury, inflammatory cytokines, and metabolic disturbance, resulting in reversible intramyelinic swelling rather than irreversible tissue infarction [3,7].

In the present case, isolated splenial involvement, absence of focal neurological deficits, and complete radiologic resolution on short-interval follow-up imaging strongly favored a cytotoxic callosal lesion rather than true ischemic infarction. Extensive evaluation for arterial, venous, cardioembolic, infectious, inflammatory, and demyelinating etiologies was unremarkable, further supporting a non-ischemic mechanism. These features underscore the importance of recognizing CLOCCs as a distinct clinicoradiologic entity to avoid misdiagnosis and unnecessary long-term antithrombotic therapy.

MBD is classically described as a rare toxic-metabolic encephalopathy affecting the corpus callosum in individuals with chronic alcoholism and malnutrition [1]. More recently, acute and reversible callosal lesions have been described in patients with toxic or metabolic insults. Although these observations suggest that alcohol-related corpus callosum injury may encompass a broader clinicoradiologic spectrum than previously recognized, the relationship between these reversible lesions and classic MBD remains uncertain [2,4]. However, the precise relationship between isolated CLOCCs and MBD remains incompletely understood. While some authors consider acute reversible callosal lesions to represent an early or milder manifestation within the MBD spectrum, others regard CLOCCs as a distinct clinicoradiologic entity with diverse etiologies [2-4].

In the present case, acute binge alcohol consumption was considered the most likely precipitating factor based on the temporal relationship with symptom onset. However, a concurrent upper respiratory tract infection may also have contributed to the development of the cytotoxic lesion, as infectious triggers have likewise been reported in association with CLOCCs. Given the temporal overlap of these potential precipitants, the precise trigger cannot be established with certainty. Alcohol-related neurotoxicity may induce transient metabolic and excitotoxic disturbances within the corpus callosum, particularly in the splenium. This predilection has been postulated to relate, in part, to a relatively higher density of glutamate receptors in splenial axons, rendering this region more susceptible to excitotoxic injury and cytotoxic edema in the setting of acute toxic or metabolic insults [7,8]. Although the absence of chronic alcohol use, malnutrition, and diffuse callosal involvement argues against classic MBD, the clinicoradiologic features overlap with those reported in acute reversible alcohol-related callosal injury. Therefore, this case is best interpreted as a possible alcohol-related CLOCC with imaging features overlapping the proposed MBD spectrum rather than definitive classic MBD. Given the patient’s young age, absence of chronic alcohol use or malnutrition, and complete radiologic resolution, MBD remains a speculative rather than definitive diagnostic consideration.

A limitation of this case is that microbiologic testing to establish a specific bacterial or viral etiology of the concurrent tonsillopharyngitis was not performed. Consequently, although an infectious trigger remains a plausible alternative explanation for the CLOCC, the underlying pathogen could not be determined.

This case highlights several important clinical implications. First, isolated splenial diffusion restriction in a neurologically intact patient should prompt consideration of CLOCCs, particularly in the presence of metabolic or toxic triggers. Second, acute binge alcohol consumption may represent one potential precipitating factor for reversible callosal lesions in young, previously healthy individuals, although concurrent infectious triggers should also be considered when present. Finally, short-interval follow-up imaging plays a crucial role in confirming reversibility and refining diagnosis in suspected stroke mimics.

To our knowledge, based on a review of the published English-language and Philippine medical literature, this represents the first locally documented case of an acute reversible CLOCC temporally associated with binge alcohol consumption in the Philippines. Increased awareness of this entity may facilitate accurate diagnosis, appropriate management, and improved patient counseling in similar clinical scenarios.

## Conclusions

This case illustrates an acute, reversible cytotoxic lesion of the corpus callosum (CLOCC) following binge alcohol consumption in a young, previously healthy individual. Isolated splenial involvement with complete radiologic resolution and an unremarkable etiologic workup favored a reversible toxic-metabolic process rather than true ischemic infarction. Although acute binge alcohol consumption was considered the most likely precipitating factor, a concurrent upper respiratory tract infection remained a plausible alternative trigger. Recognition of CLOCCs and their potential overlap with other reversible callosal injury syndromes, including the proposed alcohol-related Marchiafava-Bignami disease (MBD) spectrum, is important to facilitate accurate diagnosis, avoid unnecessary long-term antithrombotic therapy, and improve patient counseling in similar clinical scenarios.

## References

[1] Dong X, Bai C, Nao J (2018). Clinical and radiological features of Marchiafava-Bignami disease. Medicine (Baltimore).

[2] Zhang YL, Ran C, Xu C, Li W (2023). Clinico-radiologic subtypes and therapeutic observation of acute Marchiafava-Bignami disease. Sci Rep.

[3] Starkey J, Kobayashi N, Numaguchi Y, Moritani T (2017). Cytotoxic lesions of the corpus callosum that show restricted diffusion: mechanisms, causes, and manifestations. Radiographics.

[4] Hillbom M, Saloheimo P, Fujioka S, Wszolek ZK, Juvela S, Leone MA (2014). Diagnosis and management of Marchiafava-Bignami disease: a review of CT/MRI confirmed cases. J Neurol Neurosurg Psychiatry.

[5] Li S, Sun X, Bai YM (2015). Infarction of the corpus callosum: a retrospective clinical investigation. PLoS One.

[6] Garcia-Monco JC, Cortina IE, Ferreira E, Martínez A, Ruiz L, Cabrera A, Beldarrain MG (2011). Reversible splenial lesion syndrome (RESLES): what's in a name?. J Neuroimaging.

[7] Moors S, Nakhostin D, Ilchenko D, Kulcsar Z, Starkey J, Winklhofer S, Ineichen BV (2024). Cytotoxic lesions of the corpus callosum: a systematic review. Eur Radiol.

[8] Hayashi K, Nakaya Y, Ueda M (2024). Cytotoxic lesions of the corpus callosum (CLOCCs) related to hypoglycemia: clinical insights and pathogenesis. Cureus.

